# Diagnostic accuracy of Dixon water fat suppression coronary artery magnetic resonance angiography at 3.0 Tesla

**DOI:** 10.1186/1532-429X-18-S1-Q11

**Published:** 2016-01-27

**Authors:** David P Ripley, Maryam Nezafat, James R Foley, Peter P Swoboda, Markus Henningsson, Migel Vieira, Tarique A Musa, Laura E Dobson, Pankaj Garg, Bara Erhayiem, Adam K McDiarmid, Sven Plein, Rene M Botnar, John P Greenwood

**Affiliations:** 1 grid.9909.90000000419368403Multidisciplinary Cardiovascular Research Centre (MCRC) & Leeds Institute of Cardiovascular and Metabolic Medicine, University Of Leeds, Leeds, United Kingdom; 2 King's College London, Division of Imaging Sciences and Biomedical Engineering, London, United Kingdom

## Background

X-Ray coronary angiography (XRA) remains the gold standard for assessment of coronary artery disease (CAD) although has associated clinical risk and financial cost. Coronary artery magnetic resonance angiography (CMRA) requires effective fat suppression as the epicardial vessels are embedded within fat. The aim of this study was to assess the diagnostic performance of non-contrast enhanced whole-heart two-point Dixon multi-echo fat-water separation coronary angiography at 3T in patients with suspected CAD.

## Methods

Prospective, consecutive patients (n = 45) with angina underwent both XRA and free breathing Dixon 3D whole-heart CMRA within 60 days (3T Philips Achieva TX). Imaging parameters included field-of-view = 300 × 300 × 100 mm^3^, resolution = 1.2 × 1.2 × 1.2 mm^3^, TR/TE_1_/TE_2_ = 4.0/1.36/2.4 ms, α = 20°, and SENSE = 2 with a nominal scan time of 7:03 mins assuming a heart rate of 60 bpm. Navigator gating window was 5 mm with mid-diastolic image acquisition. A double-blind analysis of the two diagnostic procedures was performed at two independent centres. CMRA image quality score, vessel sharpness and length were measured and percentage diameter stenosis graded visually on a 4-point scale (4 = best). XRA stenoses were measured quantitatively (QCA).

## Results

CMRA and XRA were completed in 42(93%) patients, with a prevalence of XRA defined significant CAD of 52%. 199 of 210 (95%) proximal and mid-vessel segments were assessable with a mean image quality score of 2.7 ± 1.2, vessel sharpness 44 ± 7% and length 56 ± 23 mm. The sensitivity for the detection of CAD was 90.9% (95%CI:70.8-98.9%), specificity 75.0%(50.9-91.3), positive predictive value 80.0%(59.3-93.2), negative predictive value 88.2%(63.6-98.5), accuracy 83.3%(72.1-94.6), Likelihood Ratio Positive (LR+) 3.63(1.68-7.86) and Likelihood Ratio Positive (LR-) 0.12(0.03-0.47)(Table 1).

**Table 1:** Diagnostic accuracy of mDIXON coronary MRA for the detection of significant coronary artery disease compared to the reference test X-ray angiography.

Comparison Basis	Sensitivity (95%CI)	Specificity (95%CI)	PPV (95%CI)	NPV (95%CI)	Accuracy (95% CI)	LR+ (95% CI)	LR- (95% CI)
Per Patient (n = 42)	90.9 (70.8, 98.9)	75.0 (50.9, 91.3)	80.0 (59.3, 93.2)	88.2 (63.6, 98.5)	83.3 (72.1, 94.6)	3.63 (1.68, 7.86)	0.12 (0.03, 0.47)
Per Vessel (n = 126)	86.5 (71.2, 95.5)	90.4 (81.9, 95.8)	80.0 (64.3, 91.0)	93.8 (86.0, 97.9)	89.2 (83.6, 94.7)	8.97 (4.59, 17.55)	0.15 (0.07, 0.34)
Per Segment (Proximal and Mid Vessel Segments) (n = 196)	83.3 (67.2, 93.6)	93.0 (87.9, 96.5)	73.2 (57.1, 85.8)	96.1 (91.7, 98.6)	91.2 (87.3, 95.3)	12.0 (6.65, 21.6)	0.18 (0.09, 0.37)
Per Segment (All Segments) (n = 267)	79.5 (63.5, 90.7)	95.0 (91.2, 97.5)	73.8 (58.0, 86.1)	96.3 (92.9, 98.4)	92.7 (89.5, 95.8)	15.9 (8.75, 28.9)	0.22 (0.12, 0.44)

MRA - magnetic resonance coronary angiography; PPV - Positive Predictive Value; NPV - Negative Predictive Value; LR+ - Likelihood Ratio Positive; LR- - Likelihood Ratio Negative.

## Conclusions

Two-point Dixon CMRA at 3T detects XRA defined coronary artery disease with a sensitivity of 90.9% and specificity of 75.0%. The Dixon technique is a useful rule-out test for significant CAD with a negative predictive value of 88.2% and LR- of 0.12.

